# Spatial and Temporal Distribution of Rodents during the Epizootic and Enzootic Periods of Plague, with a Focus on Exu, Northeastern Brazil

**DOI:** 10.3390/tropicalmed6040195

**Published:** 2021-11-08

**Authors:** Diego Leandro Reis da Silva Fernandes, Matheus Filgueira Bezerra, Bruna Mendes Duarte, Mayara Paes de França Silva, Hadassa de Almeida Souza, Elainne Christine de Souza Gomes, Alzira Maria Paiva de Almeida

**Affiliations:** 1 Department of Microbiology, Institute Aggeu Magalhães—Fiocruz PE, Recife 50740-467, Brazil; leanreis7@gmail.com (D.L.R.d.S.F.); matheus.bezerra@fiocruz.br (M.F.B.); duartemendesbruna@gmail.com (B.M.D.); yara-paes@hotmail.com (M.P.d.F.S.); ahadassalmeida@gmail.com (H.d.A.S.); 2 Department of Parasitology, Institute Aggeu Magalhães—Fiocruz PE, Recife 50740-467, Brazil; elainne.gomes@fiocruz.br

**Keywords:** Rodentia, plague, *Yersinia pestis*, zoonoses, disease reservoirs

## Abstract

The plague caused by the *Yersinia pestis* bacterium is primarily a flea-transmitted zoonosis of rodents that can also be conveyed to humans and other mammals. In this work, we analyzed the spatial and temporal distribution of rodent populations during epizootic and enzootic periods of the plague in the municipality of Exu, northeastern Brazil. The geospatial analyses showed that all the rodent species appeared through the whole territory of the municipality, with different occurrence hotspots for the different species. Important fluctuations in the rodent populations were observed, with a reduction in the wild rodent fauna following the end of a plague epizootic period, mostly represented by *Necromys lasiurus* and an increase in the commensal species *Rattus rattus*. A higher abundance of rats might lead to an increased exposure of human populations, favoring spillovers of plague and other rodent-borne diseases. Our analysis highlights the role of wild rodent species as amplifier hosts and of commensal rats (*R. rattus*) as preserver hosts in the enzootic period of a specific transmission infection area.

## 1. Introduction

The plague caused by the *Yersinia pestis* bacterium is primarily a flea-transmitted zoonosis of rodents, the main hosts, that can also be conveyed to humans and other mammals [1]. Rodents constitute the most diverse order (Rodentia) of mammals, with almost 2600 species, representing 40% of the living mammal species [2]. Out of these, 279 species have already been found to be naturally infected by *Y. pestis* [3].

The plague caused three worldwide pandemics in the Christian era, claiming numerous lives, having a major impact on the course of our history, scientific development and culture [4,5]. The infection reached Brazil by sea in 1899, during the third pandemic, through the port of Santos, São Paulo state. The infection initially afflicted the brown rat population of *Rattus norvegicus* in seaports and the commensal species (*Rattus rattus*) in the rural zones of the Northeast Finally, it encountered susceptible autochthonous wild or sylvatic fauna and established several natural foci where the ecological conditions were suitable for its persistence [6,7]. These foci persisted until the present day, spreading through several mountain ranges and plateaus across the states of Ceará, Piauí, Rio Grande do Norte, Paraíba, Pernambuco, Alagoas, Bahia, Minas Gerais and Rio de Janeiro [8,9].

By analyzing the records of human plague in the Brazilian plague foci, the municipality of Exu located in the Pernambuco State, Northern Brazil, was considered the epicenter of the focal area of Chapada do Araripe [10]. Based on the concepts of a natural-permanent focus and the telluric conservation of the plague bacillus inside the rodents’ burrows, Baltazard [10] hypothesized that the plague activity would persist for longer there and reduce gradually to basal, undetectable levels, until reappearing in the same regions. Indeed, the Kernel density analysis (KDE) of the number of cases reported in Pernambuco revealed that the municipality of Exu is at higher risk for the occurrence of plague. Exu appeared at the epicenter of the Kernel patch, which radiates in decreasing intensity as it moves away from the plateau slope towards the plains and neighboring municipalities [11].

The studies on the Rodentia and Siphonaptera faunas have become an important part of the plague control program activities and several field and laboratory studies have been carried out to understand the possible role of the different rodent and flea species in the maintenance, epizootization, and epidemization of plague in the Brazilian focal areas. It is worth noting that an important part of this work was the continuous trapping of rodents to detect plague activity among their wild species, especially *Necromys lasiurus* [10,12,13,14,15,16,17].

Here, we analyzed the spatial and temporal distribution of rodent populations in the municipality of Exu, northeastern Brazil, from 1966 to 2005, during epizootic and enzootic periods of plague in the region.

## 2. Materials and Methods

### 2.1. Study Area

**Figure 1:**
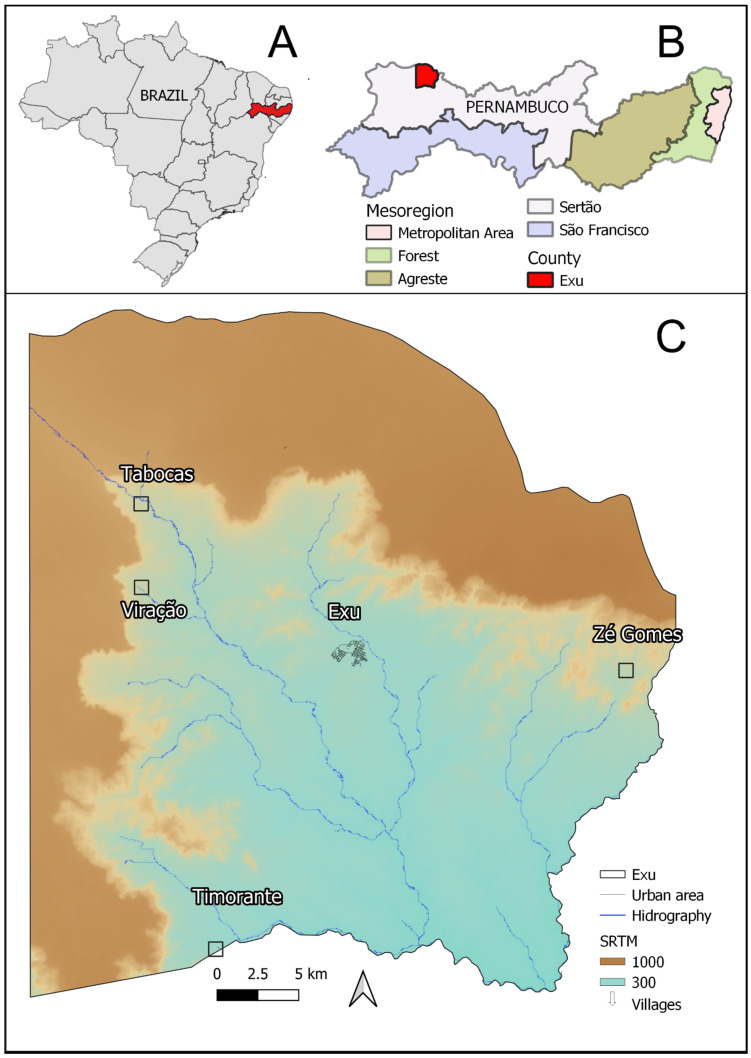
Identification of the study area: Exu, Pernambuco-Brazil. (**A**) Map of Brazil showing the state of Pernambuco highlighted in red. (**B**) Map of Pernambuco showing the mesoregions and the municipality of Exu highlighted in red. (**C**) Map of the municipality of Exu with hydrography and altitude (m), the urban area of Exu city (in the center) surrounded by other smaller rural settings the villages: Tabocas, Viração, Timorante and Zé Gomes. The shapefile of Exu was obtained from IBGE, the DEM from SRTM available online: http://www.dsr.inpe.br/topodata/ (accessed on 1 April 2021) and the hydrography from CPRM. These images are used for illustrative purposes only.

The study was performed in the municipality of Exu (Figure 1C), State of Pernambuco (Figure 1B), Northeast Brazil (Figure 1A). This municipality lies in the mesoregion of *Sertão*; it encompasses an area of 1,336,788 km², contains an estimated population of 31,825 inhabitants (according to data from 2019) and scored 0.576 on the Municipal Human Development Index (2010). Its climate is warm and dry, with scarce and irregular rainfall (Biome *Caatinga*). Situated in the ecological complex of Chapada do Araripe, 600–700 m in altitude, about 200 km long and 30 km wide, it is bordered by the municipalities of Bodocó to the west, Granito to the south, Moreilândia to the east and to the north with Crato in the state of Ceará.

### 2.2. Data Collection

The data on the rodent collection was obtained by consulting the original documents available at the Nacional Reference Service of Plague (*Serviço de Referencia Nacional de Peste: SRP*) from the Institute Aggeu Magalhães (IAM), FIOCRUZ PE, located in Recife, PE, Brazil.

The collection of rodents and fleas was performed in order to follow the *Y. pestis* circulation in the focus area over the years. The animal capture and handling methods varied according to the recommendations in each period, and further details can be found in the original publications. In short, the rodent and flea collection was carried out overnight, using rodent live traps (Chauvancy, Tomahawk, and Sherman); the trapped animals were brought to a field processing site for the collection of ectoparasites, sexing, and identification to species or genus, then they were either kept in quarantine until death or euthanized [18,19,20,21,22,23]. In the period between 1966 and 1995, only the data about the number of animals collected per year in the municipality was available, without specifying the locality. From 1996 onwards, the localities of the collections became available. The localities were georeferenced in loco, with a GPS (Global Positioning System) receptor, model eTrex Vista Cx, Garmin (Kansas City, MO, USA), configured in a DatumWGS-84. A Landmark (house, church or gate) was standardized in order to georeference each of the localities.

For geospatial analyses, the vector data obtained were the municipal limits of Exu (2010) from the Brazilian Institute of Geography and Statistics (IBGE) [24]. The drainage (hydrography) was from the Mineral Resources Research Company (CPRM) [25]—*Instituto Nacional de Pesquisas Espaciais* (INPE). The Digital Elevation Model (DEM) data was obtained from the Shuttle Radar Topography Mission (SRTM) using the script on the Google Earth Engine (GEE) platform [26]. All the geospatial data were obtained from free access and use platforms.

### 2.3. Data Analysis

**Figure 2:**
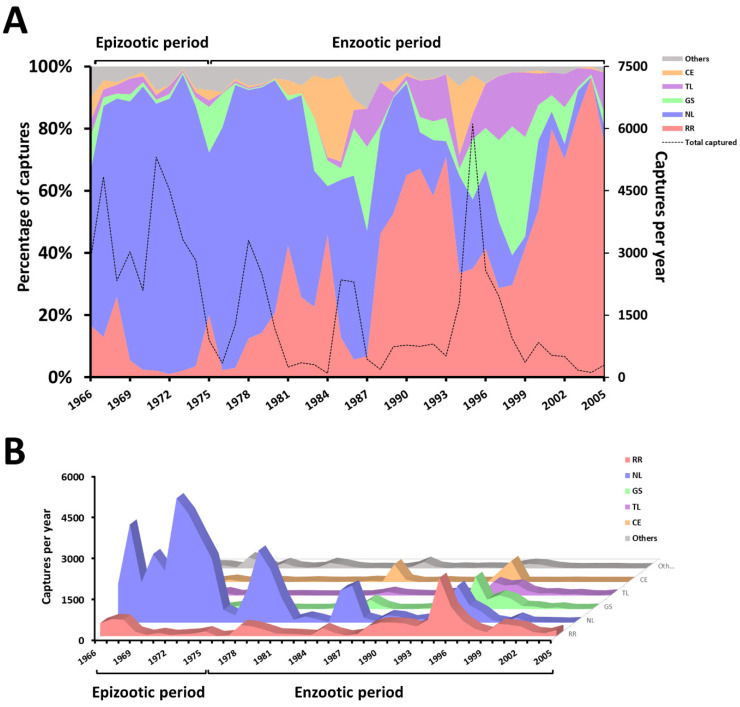
Abundance of rodents captured in Exu, Pernambuco, Brazil, 1966 to 2005. (**A**) The left axis displays the fluctuations in the proportion of captured rodent species as percentages, while the right axis shows the total number of captured animals per year. (**B**) The absolute number of captured animals per year according to the species.

**Figure 3:**
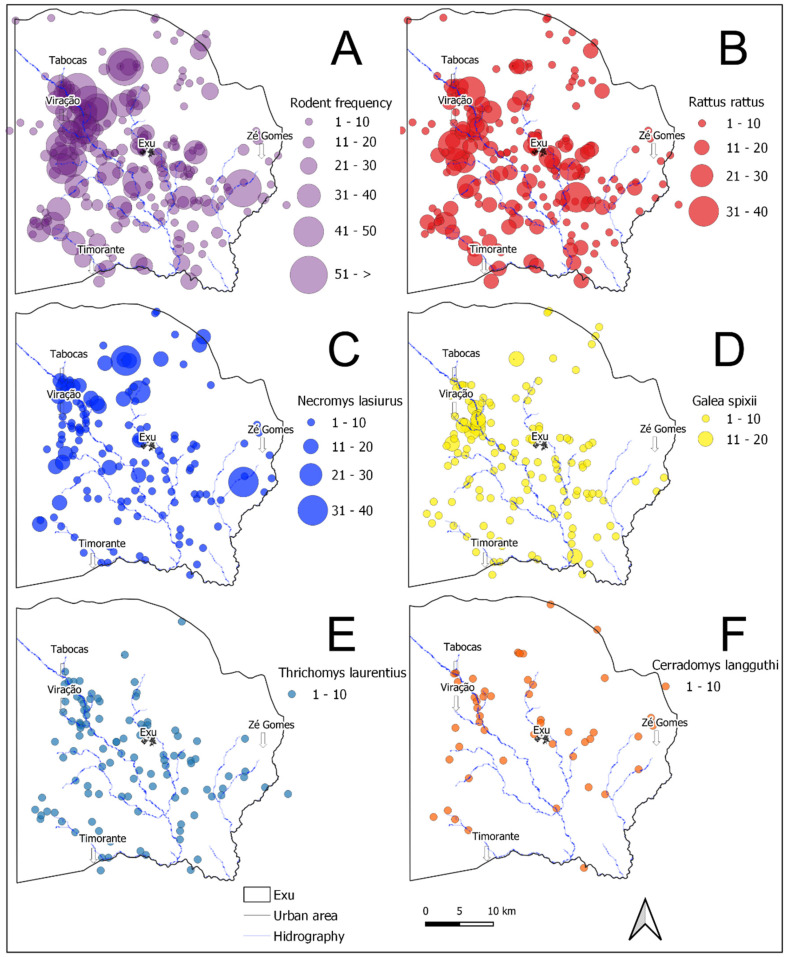
Distribution and abundance maps of the rodent species captured in the localities of Exu, Pernambuco, Brazil, 1996 to 2005. (**A**) Total rodents captured. (**B**) *R. rattus*. (**C**) *N. lasiurus*. (**D**) *Galea spixii*. (**E**) *T. laurentius*. (**F**) *C. langguthi*.

**Figure 4:**
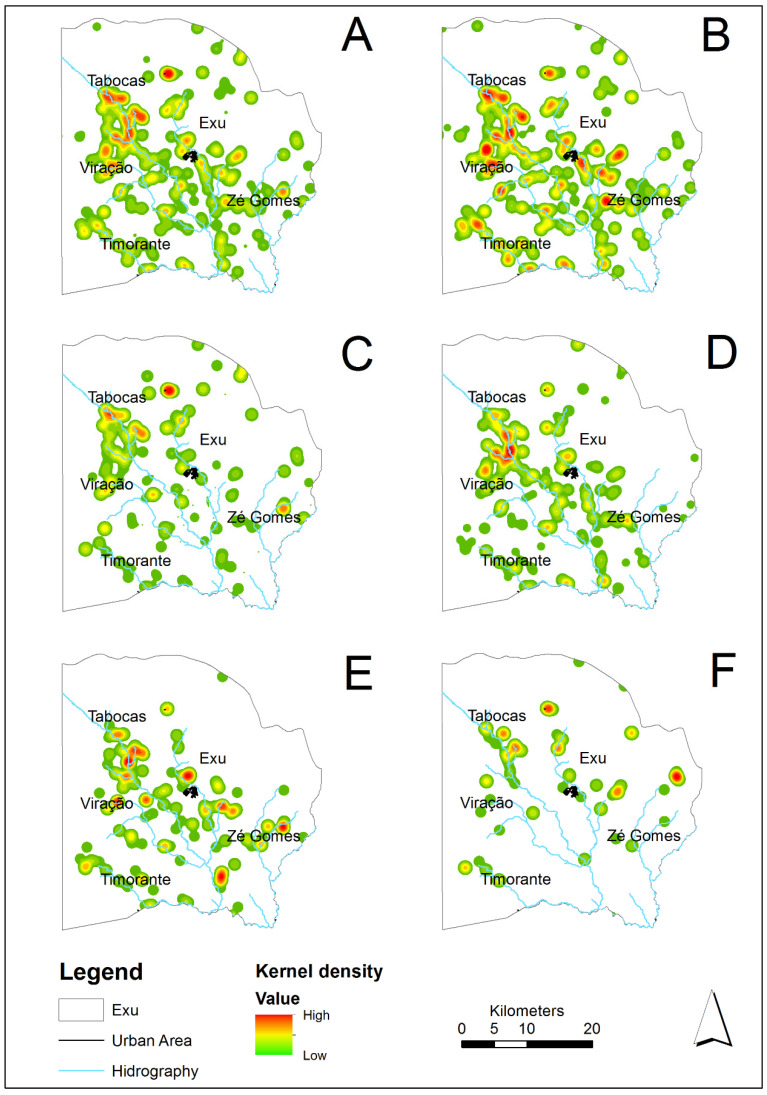
Risk maps for occurrence and density of rodent species based on the Kernel density estimator, in the localities of Exu, Pernambuco, Brazil, 1996 to 2005. (**A**) Total rodents captured. (**B**) *R. rattus*. (**C**) *N. lasiurus*. (**D**) *Galea spixii*. (**E**) *T. laurentius*. (**F**) *C. langguthi*.

The rodent species, the locality and the year of their collection were compiled and organized into a database (DB) using Excel software. While the analysis demonstrating the fluctuation of the rodent species from 1966–2005 comprised all the samples in the DB (Figure 2, *n* = 66,700), only the subset with data available on the location of the captures were included in the spatial analysis (Figure 3 and Figure 4, *n* = 3724).

The GPS data was transferred to a GPS TrackMaker Pro 4.9.603 (Geo Studio Technology, Belo Horizonte, Brazil) and the geographic coordinates were organized and stored in comma-separated values (CSV) and the shapefile format, which was then used to create the spatial database (SDB).

The spatial analyses performed were: (1) a map of the spatial distribution and abundance of the rodents to spatially visualize the localities and the number of animals collected in each locality (*sitio*, farm, village) of the municipality of Exu (choropleth maps); (2) Kernel density estimation (KDE) to identify the localization of clusters of animal occurrences. For the KDE, the following parameters were used: the bilinear interpolation method; the data classification method ‘Natural breaks (Jenks)’, with nine classes; grid cell size (bandwidth method), defined using an adaptive radius—as it is more applicable to the use of data from animals with different dispersion radii—with the area unit defined in m^2^. The choroplethic maps of the localization and density of the rodents collected were produced by the software Qgis Desktop 3.16.5 [27]. The Kernel maps built with ArcGIS 10 [28].

## 3. Results

### 3.1. Fluctuation in the Abundance of Rodent Species from 1966 to 2005

Through long-term monitoring (1966–2005) of the plague activities in the municipality of Exu (Figure 1A–C), 66,700 rodents from eight species were captured: *Necromys lasiurus* (=39,797), *Rattus rattus* (=13,132) *Galea spixii* (=4581), *Thrichomys laurentius* (=3195), *Calomys expulsus* (=2696), *Cerradomys langguthi* (=2481), *Oligoryzomys nigripes* (=680) and *Wiedomys pyrrhorhinos* (=138). Figure 2 shows the fluctuation in the abundance of rodent species in percentage (2A) and absolute values (2B) per year, over the 40-year’ period between1966 and 2005.

The species *N. lasiurus* and *R. rattus* were the most abundant throughout the study period (1966 to 2005). Until 1987, the rodent *N. lasiurus* was the predominant species (≅40 to 97% of the catches) but from 1988 onwards the rat (*R. rattus*) became predominant (≅28 to 96% of the catches), while the number of *N. lasiurus* decreased to 0–37% of the catches. The species *W. pyrrhorhinos* and *C. expulsus* occurred constantly in basal numbers and from 1990 onwards, no *O. nigripes* were captured (Figure 2A,B).

Due mostly to the reduction in the *N. lasiurus* population, there was a substantial decline in the overall number of captured animals between 1966 and 1981. However, population spikes were observed during the intercalated periods of 1985–1986 and 1994–1997. Notably, with the exception of *W. pyrrhorhinos* and *O. nigripes*, there was an increase in the total number of most species captured from 1994 to 1997 (Figure 2A,B; Supplementary Table S1).

### 3.2. Spatial Distribution of the Rodents’ Populations in the Period Analyzed (1996–2005)

Regarding the geographical distribution in the period analyzed (analysis limited to 1996–2005), all the species occurred in the same *sitios* or farms scattered through the whole territory of the municipality (Figure 3A and Figure 4A). Figure 3B–F and Figure 4B–F show the spatial distribution and frequency hotspots of the species *R. rattus*, *N. lasiurus*, *G. spixii*, *T. laurentius* and *C. langguthi* from 1996 to 2005. Due to the small quantity in this period, the species *C. expulsus* (=15), *W. pyrrhorhinos* (=23) and *O. nigripes* (=0) were not included in the maps.

The *R. rattus*, the most abundant species found during the period (1966–2005), was widely disseminated throughout the territory and occupied a higher number of localities (Figure 3B). However, the areas with the highest density and considered hotspots for the occurrence of this species were in the boundaries of the villages Tabocas, Viração, Timorante and Zé Gomes (Figure 4B). *N. lasiurus* was also found throughout the territory and presented several hotspots near the villages Tabocas and Viração, as well as aa hotspot near the village of Zé Gomes (Figure 4C). The relatively abundant population of *G. spixii* was also disseminated throughout the territory and presented hotspots in the boundaries of the villages Tabocas and Viração, in the southern part of the municipality, as well as another hotspot on the plateau of the Chapada do Araripe (Figure 4D). The *T. laurentius* hotspots occurred in the boundaries of the villages Tabocas and Viração and of the city of Exu and others in the southeast of the municipality (Figure 4E). *C. langguthi*, the least numerous species and with the lowest dispersion (Figure 4F), presented a distribution of hotspots different from the others occurring along the slope of the Chapada do Araripe and in the boundaries of the village Zé Gomes.

**Figure 5:**
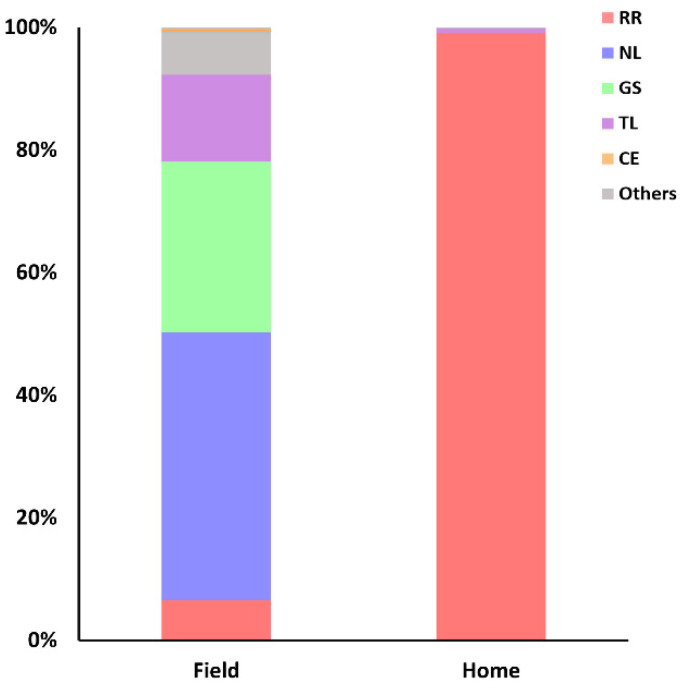
Proportion of species captured in field or household environments.

Importantly, marked differences were observed in captures from traps set at household or field environments. The proportion of traps set in fields or household environments was standardized in 3:1, respectively. While the proportions of *N. lasiurus* and *R. rattus* in field captures were 44% and 6.5%, respectively, 99% of household captures were *R. rattus* and no *N. lasiurus* were found in this environment (Figure 5).

## 4. Discussion

Practically since the arrival of the plague in Brazil in 1899, during the third pandemic, a surveillance and control program adjusted to the epidemiological situation, ecological and demographic characteristics and scientific and technological conditions has been carried out [7,10,17]. For several decades, the rodents were trapped for the detection of the plague bacillus and/or anti-plague antibodies [22,23,29]. The surveys among the rodents were discontinued in 2007 due to new evidence that the serological survey of plague antibodies among roaming dogs is a more efficient and cost-effective tool for plague surveillance [17,20]. By compiling the data from 40 years (1966 to 2005) of monitoring in the plague focus region of Chapada do Araripe, we were able to observe an important fluctuation in the number of captured rodents (Figure 2A,B). It is important to highlight that while the period that saw the predominance of *N. lasiurus* comprises the years in which human cases of plague were noted in the region (1966–1976), the period that saw the predominance of *R. rattus* overlapped with the enzootic period of plague [17].

Rodent populations are known to undergo significant fluctuations over both seasonal and multiannual cycles, which also impacts on the risk of zoonosis spillovers to humans [30,31]. Here, we observed four-to-seven-year intervals in the pendular *N. lasiurus* population spikes. However, from the last years of human cases of plague onwards, their abundance peaks progressively decreased both in frequency and abundance. From the 1995 peak until the end of the study period (2005), no *N. lasiurus* population growths were observed. The decline of these populations might have been due to the important and continuous plague deaths of susceptible species over many years, climate change and environmental alterations created by agriculture [9,17,32].

From 1996 to 2005, no *O. nigripes* were observed and *C. expulsus* and *W. pyrrhorhinos* were captured in small numbers (Figure 2A,B). The reduction or disappearance of these species does not qualify them as endangered species at risk of extinction because this is only a local event [30,33]. It is noteworthy that some species may multiply suddenly and explosively, a phenomenon popularly known as “ratadas”. This phenomenon is generally correlated with an unusual availability of specific food that occurred in the State of Bahia, involving the species *W. pyrrhorinos* in 2002 and *C. expulsus* in 2015 [34].

As observed in Figure 3A and Figure 4A, all the species appeared in the whole territory of the municipality. The wild species lived off agricultural products, which they consumed in situ. Although occupying the same places (*sitios* or farms) dispersed throughout the territory, the different species did not occupy the same habitats. The species *N. lasiurus*, *C. expulsus*, *C. langguthi*, *O. nigripes* and *W pyrrhorhinos* usually shelter in sites covered by low and dense vegetation, where they make their nests. Besides, *C. langguthi*, *O. nigripes* and *W. pyrrhorhinos* can make nests in small trees or rock walls. Others (*G. spixii*, *T. laurentius*) shelter in the cracks and crevices of rocks, further away from humans [10,34]. Along with field observations, these results are not suggestive of attraction or avoidance patterns, with implications for competitive relationships and plague transmission among these species [35].

The main economic activity in practically all the rural land of the municipality of Exu is dedicated to agriculture practiced in the “*sitios*”, which are mainly located along the hydrographic network on the slopes of the Chapada do Araripe (seen in the satellite image in Figure 1C), where remnants of native vegetation (*caatinga*) are also found. The term “*sitio*” means a rural land division usually including housing, functional buildings (barns, garages, storage areas) and a parcel for cultivating and/or raising stock. The human dwellings are generally unpaved or cemented or composed of brick floors, clay or brick walls and a roof of tile, zinc, grass or straw. They are often used as both housing and storage for crop products (maize, beans and cotton grains). Unlike the *R. rattus*, wild rodents rarely enter these dwellings.

The urbanization of some rural communities living with precarious sanitary infrastructure has created the ideal conditions for the expansion of the commensal rat [36]. Therefore, *R. rattus* were more abundant inside household captures (Figure 5) or in the boundaries of the villages Tabocas, Viração, Timorante and Zé Gomes.

The high abundance of rats in these villages might lead to more contact between them and the inhabitants, favoring plague and other rodent-borne diseases [37]. Therefore, some preventive measures should be implemented in these villages, including surveillance and rodent and insect control [20]. Commensal rat (*R. rattus*) control includes educative measures for proper grain storage; eliminating rats by clearing the land around houses, thereby making the environment unsuitable for them; and rat extermination, using rodenticides [7,20]. Flea control was carried out using the insecticides DDT (Dichlorodiphenyltrichloroethane) and BHC (Benzene hexachloride), which unfortunately led to the selection of resistance, by continuous pressure, of rat fleas (*Xenopsylla cheopis*) and human fleas (*Pulex irritans*) explaining the ineffectiveness of preventive measures based on the continuous use of these insecticides [10,11,16,34].

In a previous study in this same plague area, the transition of the infection from urban to rural areas was observed [11]. The plague reappeared in rural areas after a six-year inter-epizootic period and disseminated among the wild fauna practically throughout the municipality territory. According to Figure 3A–F, the dispersal area of the rodents in the present study overlapped with the sites of the distribution of the human cases shown by Fernandes et al. [11].

The plague disappeared suddenly in this focus area from 1975 [11]. This may have been associated with the rarefaction of the susceptible species, mainly the population of *N. lasiurus*, which is considered the amplifier host. In spite of the increase in the *R. rattus* population during the 1990′s, plague activity was no longer detected in rodents or humans, as *Y. pestis* bacterium was last isolated in 1987, and serologic testing for anti-plague antibodies in sentinel animals has declined over time [17].

Rats are relatively resistant to fatal plague infection and do not suffer from major death rates that could lead to epizootization in the absence of susceptible species [10,15,38,39]. On the other hand, the *R. rattus* might act as a preserver host by keeping plague dormant until eventual flea re-infection reactivates the epizootic cycle after the restoration of susceptible wild hosts populations [3,32,40].

The *X. cheopis* is the prevalent flea among *R. rattus* that may harbor wild rodent fleas, but in small numbers [16]. The wild rodent species primarily harbor two species of fleas (*Polygenis bohlsi jordani* and *Polygenis tripus*) that likely play an important role in plague spread in the ecosystem and the occurrence of human cases. Previous results established the species *N. lasiurus* and its fleas as the epizootic amplifier hosts, spreading the infection to other species, even less susceptible ones, such as the commensal rat, eventually causing spillovers to human populations [10].

## 5. Conclusions

The data presented in this study highlight that *N. lasiurus* might be responsible for plague epidemics in this focal area of transmission in northeastern Brazil, since the reduction in the abundance of this species over time coincided with the enzootic period of the disease. Furthermore, the increase in the abundance of *R. rattus* is directly related to the urbanization of small rural localities. In spite of their abundance, the rats did not drive plague epidemics as might be expected, especially considering their proximity to humans. As the plague infection cycle can reactivate after several years of epidemiological silence, an enzootic period must not be misinterpreted as the extinction of a plague focus [1,5,32]. Therefore, continuous surveillance is required and preventive measures focused on driving rodents away from houses, along with protection against flea bites, should not be overlooked.

## Data Availability

The data presented in this study are available in Supplementary Materials.

## Supplementary Materials

The following are available online at https://www.mdpi.com/article/10.3390/tropicalmed6040195/s1, Table S1. Rodents captured in Exu, Pernambuco, Brazil, 1966 to 2005.
